# Synthesis of silver–palladium Janus nanoparticles using co-sputtering of independent sources: experimental and theorical study

**DOI:** 10.3762/bjnano.15.67

**Published:** 2024-07-04

**Authors:** Maria J Martínez-Carreón, Francisco Solís-Pomar, Abel Fundora, Claudio D Gutiérrez-Lazos, Sergio Mejía-Rosales, Hector N Fernández-Escamilla, Jonathan Guerrero-Sánchez, Manuel F Meléndrez, Eduardo Pérez-Tijerina

**Affiliations:** 1 CICFIM Facultad de Ciencias Físico Matemáticas, Universidad Autónoma de Nuevo León, San Nicolás de los Garza, Nuevo León, 66455, Mexico; 2 Instituto Superior de Tecnologías y Ciencias Aplicadas (InSTEC). Universidad de La Habana, San Lázaro y L, Vedado La Habana, 10400, Cuba; 3 Centro de Nanociencias y Nanotecnología, Universidad Nacional Autónoma de México, Apartado Postal 14, Ensenada, Baja California, 22800, Mexico; 4 Facultad de Ciencias para el Cuidado de la Salud, Universidad San Sebastián, Campus Las Tres Pascualas, Lientur 1457, Concepción 4060000, Chile

**Keywords:** bimetallic nanoparticles, inert gas condensation, Janus nanoparticles, silver–palladium nanoparticles

## Abstract

Janus-type nanoparticles are important because of their ability to combine distinct properties and functionalities in a single particle, making them extremely versatile and valuable in various scientific, technological, and industrial applications. In this work, bimetallic silver–palladium Janus nanoparticles were obtained for the first time using the inert gas condensation technique. In order to achieve this, an original synthesis equipment built by Mantis Ltd. was modified by the inclusion of an additional magnetron in a second chamber, which allowed us to use two monometallic targets to sputter the two metals independently. With this arrangement, we could find appropriate settings at room temperature to promote the synthesis of bimetallic Janus nanoparticles. The structural properties of the resulting nanoparticles were investigated by transmission electron microscopy (TEM), and the chemical composition was analyzed by TEM energy dispersive spectroscopy (TEM-EDS), which, together with structural analysis, confirmed the presence of Janus-type nanostructures. Results of molecular dynamics and TEM simulations show that the differences between the crystalline structures of the Pd and Ag regions observed in the TEM micrographs can be explained by small mismatches in the orientations of the two regions of the particle. A density functional theory structural aims to understand the atomic arrangement at the interface of the Janus particle.

## Introduction

Janus-type nanoparticles are specific structures that present two faces or regions with different chemical or physical properties in a single particle. Their compositional asymmetry can lead to interesting interactions with their environment or other materials, which give them properties and potential applications that homogeneous nanoparticles do not have. For instance, the distinct sides of Janus nanoparticles can be functionalized with different surface chemistries, allowing for controlled interactions with different molecules, surfaces, or biological entities; this feature may be particularly useful in applications as diverse as drug delivery, catalysis, and sensors.

The methodologies, developed for the first time in 1999 [1], for the production of bimetallic nanoparticles in the gas phase can be roughly classified as either simultaneous or sequential. In the first category, the materials that will be used to make the nanoparticles are evaporated concurrently in the aggregation region, which results in the formation of nanoparticles with a variety of structural motifs, such as randomly alloyed, core–shell, and Janus nanoparticles [2].

The study of bimetallic nanoparticles (BNPs) is highly interesting for the scientific community because of their possible technological applications in catalysis and biosensors, and within materials science in general [3]. To date, most published articles on BNPs have focused on the synthesis of miscible metals, such as PtPd or CuPd; the literature on bimetallic nanoparticles of non-miscible metals, such as AuNi and PdRu, is limited [4]. In this sense, the production of nanoalloys made of immiscible metals in volume has generated a large amount of interest because this lack of miscibility may be used as an advantage for the design of nanomaterials with new functional properties, distinct from those offered by the same two metals in the bulk, such as Au and Ni [4]. Alloying immiscible elements is feasible in the nanoscale regime because the enthalpy of the mixture decreases as the size of the nanoparticles decreases, and it generally becomes negative below a certain particle size [5].

Silver–palladium alloyed nanocrystals are currently materials of high interest in electronics, specifically because they can be used in the building of electrodes for multilayer ceramic capacitors, exhibiting better thermal performance than silver nanocrystals, partly, because Pd has a relatively high critical melting temperature and, partly, because the appearance of moisture delays the diffusion of silver atoms. The synthesis of AgPd nanocrystals with sizes ranging from 2.46 to 6.65 nm has been reported for applications in the manufacturing of electronic components [6].

Chu et al. [7] synthesized Pd–Ag nanoparticles, by the wet reduction method using palladium and silver hydroxide colloids as precursors, to study hydrogen absorption; the size of these BNPs was 6–7 nm. However, inhomogeneous nanoparticles were obtained because Ag fractions were found on the surface, which were increased by heating the samples under vacuum. AgPd nanoparticles also exhibit activity in the hydrogenation of acetylene and ethylene. Khan et al. [8] found that the addition of Ag to Pd supported on alumina suppresses the general hydrogenation activity, but it also increases the selectivity towards ethylene avoiding acetylene poisoning, resulting in an increase in the useful life of the catalyst. Chunling An et al. synthesized AgPd BNPs in aqueous solution with sizes from 4 to 5 nm and demonstrated that they exhibit greater electrocatalytic activity and better long-term performance than silver nanoparticles [9].

Janus nanoparticles, which are basically defined by two metals joined side by side, may produce significant interfacial effects [10]. Qiu et al. [11] investigated the role of the injection rate of the chemical precursors on the elemental distribution in AuPd nanoparticles. They found the injection rate needed to promote twin proliferation, which favored the production of Pd−Au Janus icosahedra. In the same experimental setup, they also promoted twin elongation, which aided the production anisotropic Pd@Au core–shell starfish-like structures. As it is known, icosahedral nanoparticles are formed by 20 tetrahedral subunits. The authors obtained a low concentration of Au atoms in one side of the Pd decahedral seed at a slow injection rate. This eventually produced an asymmetric growth mode at a slow kinetic rate, transforming the decahedron into a PdAu icosahedron with five tetrahedra rich in Pd, and the other 15 tetrahedra rich in Au [11].

This work reports the production (for the first time, to our knowledge) of Ag/Pd Janus BNPs, using the inert gas condensation (IGC) technique, where the materials are obtained from independent palladium and silver targets. In order to achieve this, the original IGC setup, as designed and built by Mantis Ltd., was modified by adding a second magnetron in a different chamber, in contrast to using a bimetallic target, as Pérez-Tijerina and coworkers did [12]. Unlike other synthesis methods, the IGC technique enabled the BNP synthesis at room temperature. Theoretical studies were carried out by molecular dynamics and TEM simulations to investigate the atomic ordering and orientation of the crystal lattice, while a detailed description of the atomic arrangement at the interface between the two metals was obtained using density functional theory (DFT).

## Experimental

In this work, the nanoparticles were obtained using a Nanosys 500 system from Mantis Ltd. modified by the addition of a second magnetron in a different chamber to obtain two nanoparticle sources, in a similar manner as described in [13]. In the first magnetron section, Ag atoms were generated from a silver target with 99.99% purity; the sputtered atoms traveled to the second chamber, where a shower of Pd atoms was generated on the second magnetron from a palladium target with 99.99% purity. The BNPs generated by condensation passed through a quadrupole mass filter for size selection and were finally deposited on holey carbon copper grids in an argon flow at 52 sscm for 5 min at room temperature.

The size of the BNPs can be modified by varying three parameters, namely, aggregation area, magnetron power, and partial pressure. The chosen parameters for both magnetrons are presented in Table 1.

**Table 1:** Synthesis conditions for bimetallic AgPd particles.

Parameter	Initial	In operation

argon flow [sccm]	60	60
voltage, first magnetron [kV]	1.000	0.297
voltage, second magnetron [kV]	0.984	0.305
temperature [°C]	10.0	9.9

HRTEM micrographs were obtained using a JEOL JEM-2100F microscope. The EDS analysis was performed using INCA software. In order to assist the interpretation of the electron micrographs, several atomistic models of AgPd nanoparticles were created. These models consist of nanospheres of approximately 10 nm diameter, for a total of 34,467 atoms arranged in a face-centered cubic (fcc) lattice. The atoms of one region of the particle were identified as Ag, while the other region was made of Pd. The model particles underwent a thermalization process with a molecular dynamics (MD) run in the canonical ensemble, at a temperature of 300 K, using the DL_POLY 4 code [14]. The time step was set to 0.001 ps, for a total simulation time of 0.5 ns. The atomic interactions were modelled using the Sutton–Chen potential, with the parameters obtained by Çağin et al. [15], and the mixing rules of Rafii-Tabar and Sutton were used to define the crossed Ag–Pd interactions [16]. The final structures were used to generate simulated TEM micrographs using the SimulaTEM package [17] and approximated STEM micrographs, with the assumption that the signal intensity, *I*, of atoms of atomic number *Z* goes as *I* = *Z^n^*, where *n* is a number close to 2 [18].

To complement the structural analysis of Janus BNPs, we performed a set of atomic-scale studies using density functional theory [19–20] as implemented in the Vienna Ab Initio Simulation (VASP) package [21–22]. VASP employs a plane wave basis set to represent the electronic states. We used the generalized gradient approximation (GGA) to compute the exchange–correlation energies using the Perdew–Burke–Ernzerhof parameterization [23]. The projector augmented wave (PAW) pseudopotentials were considered with a cutoff energy of 500 eV [24]. Geometrical optimization of all models was performed without any constraints until the forces were less than 0.02 eV/Å. To consider surface effects, we break the symmetry along the *z* axis by introducing a vacuum space of 20 Å to preclude surface self-interaction. The Brillouin zone for the 3D bulk phases was sampled with an 8 × 8 × 8 *k*-points mesh under the Monkhorst–Pack scheme [25]; 8 × 8 × 1 and 4 × 4 × 1 *k*-points, respectively, were considered for the Ag/Pd and X/AgPd (X = Ag, Pd) Janus models considering surface effects.

## Results and Discussion

The experiment was carried out under three conditions, namely, only the first magnetron section working to confirm the sputtering Ag nanoparticles, only the second magnetron section working to confirm the sputtering of Pd nanoparticles, and both magnetrons working to obtain BNPs of the desired size.

**Figure 1:**
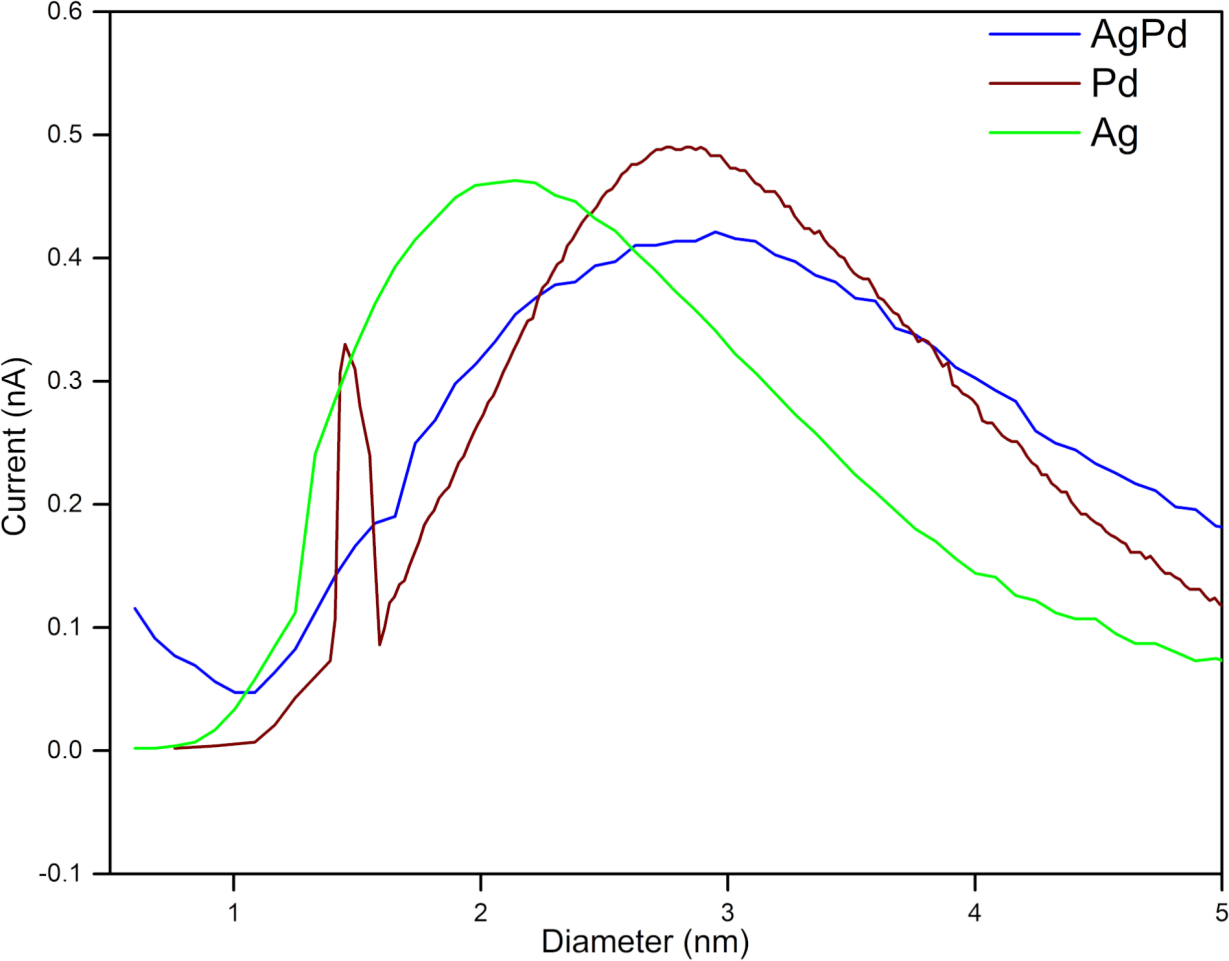
Size distribution profiles determined using a quadrupole mass filter.

Figure 1 shows the nanoparticle size distribution for each experimental condition. The green color profile corresponds to the nanoparticles sputtered only by the first magnetron section from a silver target, whereas nanoparticles sputtered only by the second magnetron from a palladium target are illustrated in red. Finally, the blue profile corresponds to the case in which the two magnetrons are working simultaneously.

In this way, we could establish the conditions to obtain the Ag/Pd nanoparticles. It is important to highlight that, by correctly selecting the deposition conditions, we can obtain either structures in Janus configurations or uniformly distributed alloys of these materials at room temperature, as will be shown later in this paper.

**Figure 2:**
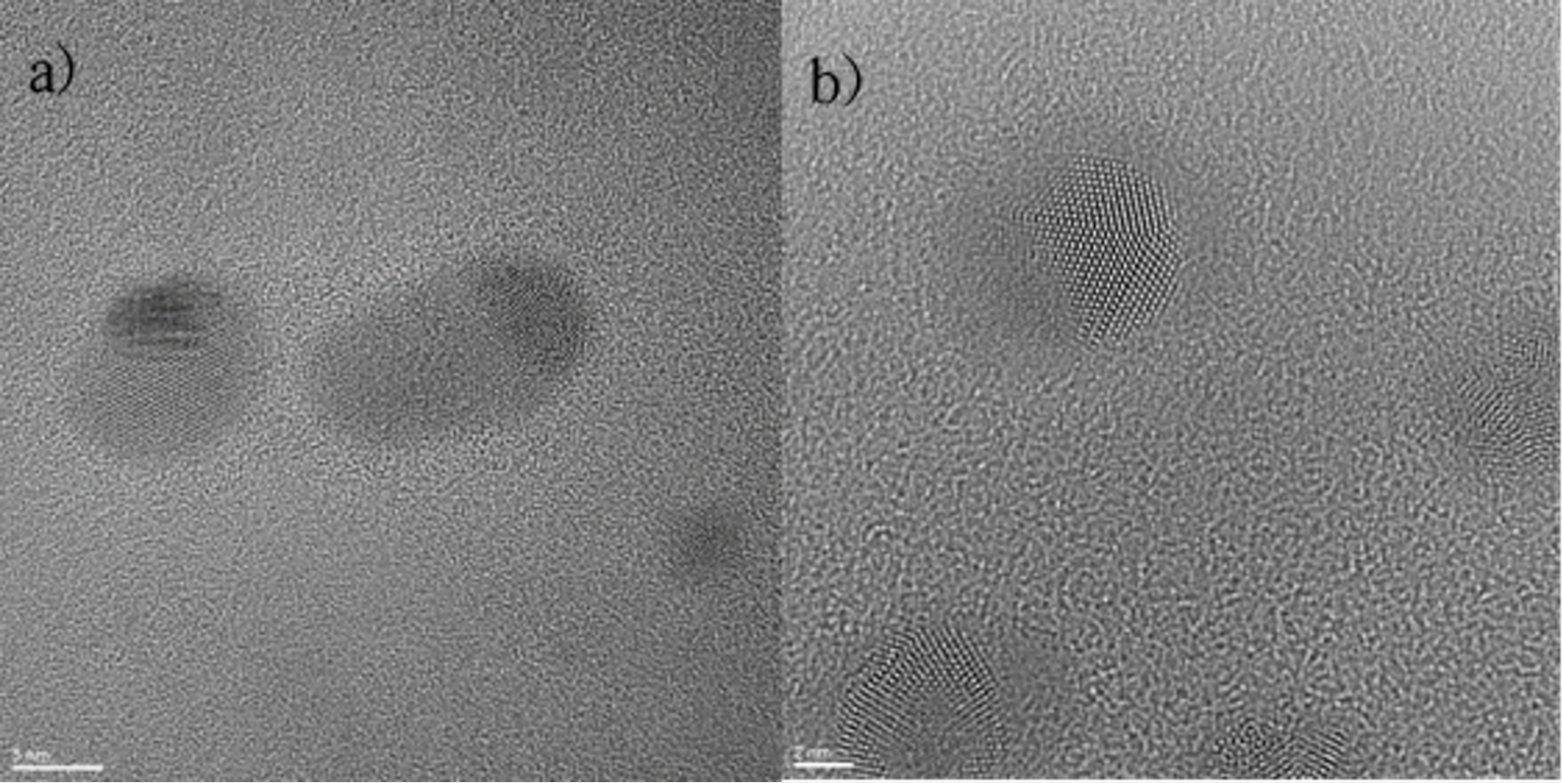
HRTEM micrographs of AgPd nanoparticles. (a) Janus-type structure. (b) Janus-type structure.

Figure 2 shows two TEM micrographs. In Figure 2a, it can be seen that the nanoparticles have a Janus-type structure. Their growth in a preferential orientation in the assembly process enabled the formation of this complex and hierarchical structure. In addition, it is observed that, in the same nanoparticle, there are two different structures. An approximation of one of these nanoparticles in Figure 2b shows more evidently that there is one structure on the surface of another in a controlled manner.

**Figure 3:**
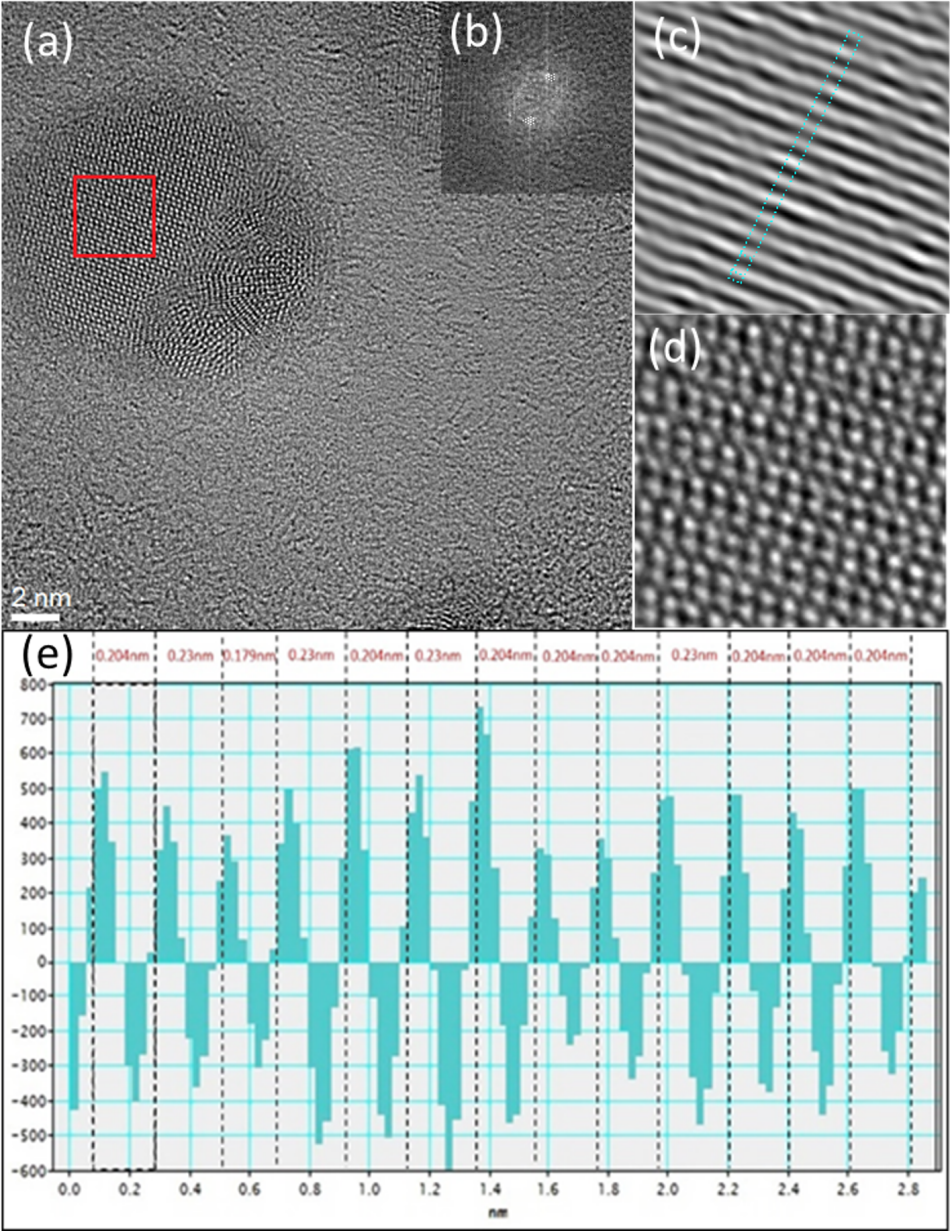
Calculation of interplanar distances in silver–palladium nanoparticles. (a) HRTEM micrograph, (b) diffraction pattern, (c) reflection image of white circles, (d) reflection image of all points in the diffraction pattern, and (e) crystallographic distance profile.

Figure 3a shows the micrograph used for the calculation of the interplanar distances. The diffraction pattern of this section is presented in Figure 3b. The image obtained from the reflection of the white circles indicated in the diffraction pattern is shown in Figure 3c, and the reflection of all points is shown in Figure 3d. These results show lattice fringes corresponding to the crystal structures of Ag and Ag–Pd, with the measurements matching known interplanar spacing values.

Interplanar distances of 0.204 and 0.230 nm were obtained using the Digital Micrograph software. According to ICDD standards, the interplanar distance of 0.204 nm corresponds to the (111) plane of a cubic Ag system with reference code 00-004-0783, whereas the interplanar distance of 0.230 nm, according to crystallographic chart 01-072-5157, corresponds to the (111) plane of a cubic system of an AgPd compound. Forming the plasma in different chambers favors this type of growth.

**Figure 4:**
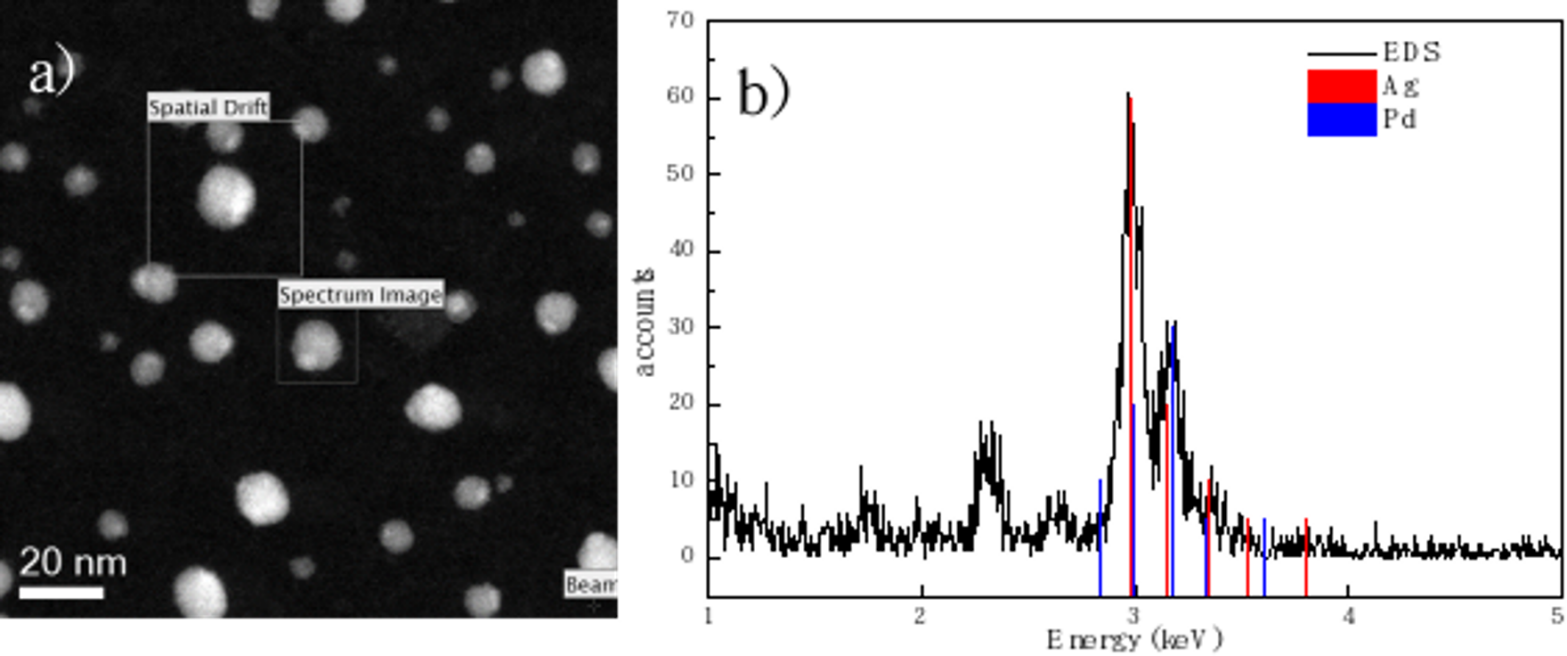
Elemental analysis by TEM-EDS of the silver–palladium nanoparticles. (a) STEM micrograph, (b) spectrum of elementary analysis.

An elemental analysis (EDS) of the silver–palladium sample was also performed on a TITAN FEI Analytical (Low-Base) device in STEM mode. Figure 4a shows the micrograph with the area in which the scan was performed is marked by the box “Spectrum Image”; the section that serves for the correction of spatial distortion has the legend “Spatial drift”. Figure 4b presents the spectrum of elementary analysis; the two elements silver and palladium appear. All characterization techniques, as well as the calculation of the interplanar distances, showed only the presence of silver and palladium without any contaminants or other metals, which validates the condensed gas technique for obtaining BNPs.

**Figure 5:**
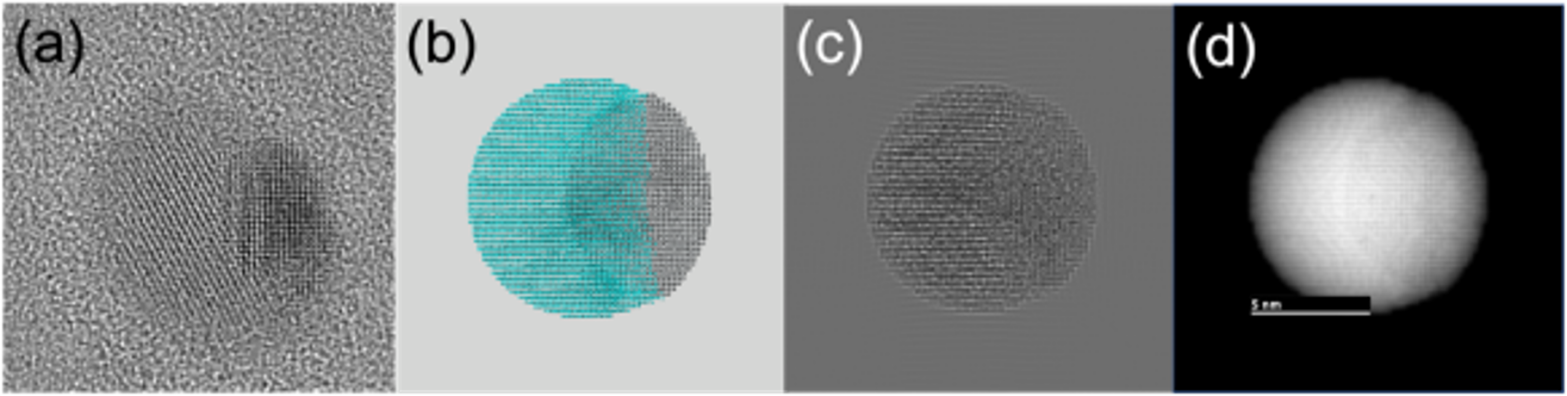
Simulated electron micrographs of a Janus AgPd nanoparticle. (a) HRTEM micrograph of a real nanoparticle. (b) Atomistic model; gray spheres represent Ag atoms, blue spheres represent Pd atoms. The Ag lattice is rotated by 5° with respect to the Pd lattice. (c) Simulated TEM micrograph of the model in (b). (d) Simulated *Z*-contrast image of the model in (b).

As an aid for the interpretation of the experimental TEM micrographs, a set of simulated micrographs of model Janus particles were generated. Figure 2 and Figure 3 show that the BNPs consist of two distinct regions, differing from each other in intensity and atomic lattice. The crystal structures of Ag and Pd are quite similar; both elements are fcc with almost the same atomic mass, but their atomic radii differ by about 12%. We built a model of Janus particles formed by an fcc spherical volume where a Pd region partially covers an Ag region and thermalized the structure at 300 K using molecular dynamics in the NVT ensemble. The atomic coordinates of the final configuration of the MD run were fed to a proprietary software to build STEM-like micrographs, and simulated TEM micrographs were created with the SimulaTEM program. The analysis of the simulated images indicates that the difference in atomic radii alone is not enough to explain the features of the real micrographs. We modified the model through rotating the Ag region by 5° to generate a small mismatch between the orientations of the lattices, performed a MD thermalization, and produced a new set of simulated micrographs. The results are shown in Figure 5. This model, represented in Figure 5b, can be compared against a real particle of similar size taken from Figure 2 and shown in Figure 5a. Figure 5c shows the simulated TEM micrograph; here, we can note that the small lattice mismatch is able to reproduce the main features of the real micrograph. Even using simplified BNP models, where both elements are completely separated from each other, unlike what appears to be the case in the real nanoparticles, it is remarkable that a small mismatch between the orientations can produce well-defined regions in the TEM micrographs. In STEM, the main differences in intensity are expected to occur when two elements with notably different atomic numbers interact with the electron beam; in this case, the two elements involved have very similar atomic numbers (46 for Pd and 47 for Ag); thus the differences in intensity in the regions of a particle are unlikely to be due to differences in atomic number. We generated images based on the assumption that the intensity has a *Z*^1.7^ dependence; only when there is a small orientation mismatch, the Pd and and Ag regions can be distinguished. An example of one of these images is shown in Figure 5d, and it can be compared against the STEM micrograph of Figure 4.

**Figure 6:**
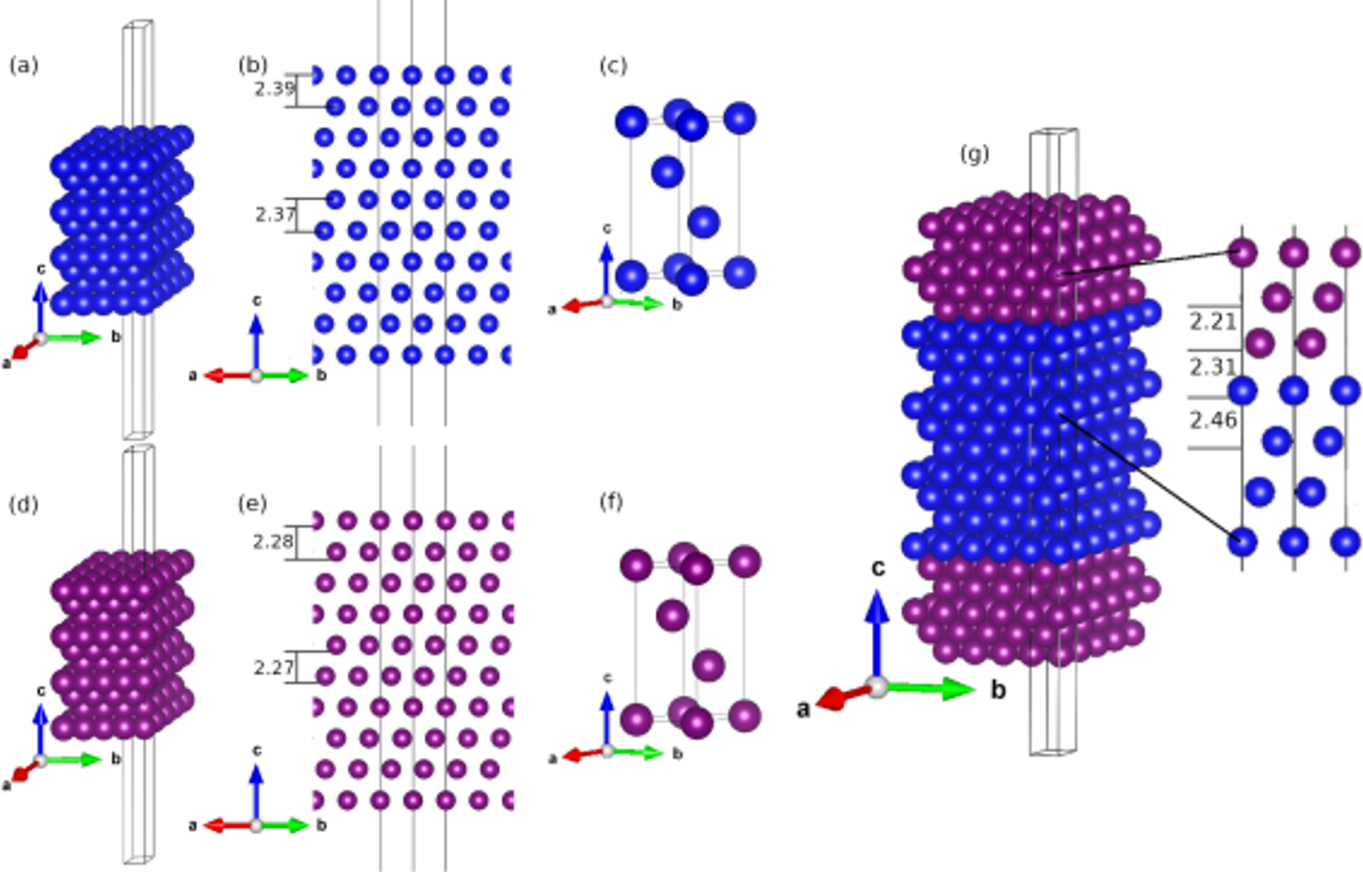
(a) Perspective view of the Ag surface space sphere filling model, (b) side view of the (111) Ag surface with the calculated (111) interplanar distance, (c) (111) Ag unit cell, (d) perspective view of (111) Pd surface space sphere filling model, (e) side view of Pd surface with the calculated (111) interplanar distance, (f) (111) Pd unit cell, and (g) the first AgPd Janus model. Blue and magenta spheres represent Ag and Pd, respectively.

By using density functional theory, we simulated the pristine Ag and Pd fcc bulk phases. The calculated lattice parameters are *a* = 4.12 Å and *a* = 3.93 Å for Ag and Pd, respectively. Because the (111) interplanar distance is reported in the Experimental section, we computed the Ag and Pd (111) surface models. The Ag(111) (Figure 6a,b) and Pd(111) (Figure 6d,e) surfaces were modeled in a hexagonal lattice, with a slab thickness equivalent to three unit cells (approximately 2 nm). All models considered inversion symmetry to save computational resources. The central unit cell is fixed to retain a bulk-like behavior. The remaining atoms are free to move without any constraints. After relaxation, the calculated (111) interplanar distances in the bulk and at the surface are, respectively, 2.37 and 2.39 Å for Ag, and 2.27 and 2.28 Å for Pd. The slight interplanar distance increment is attributed to surface effects.

Because Ag atoms are obtained at the first magnetron and Pd is available at the second magnetron, the first Janus particle considered assumes pure epitaxial growth of Pd on Ag(111). To compute this model, we deposited a Pd slab on top of a Ag slab. The lattice mismatch between them is 4.6%. In this way, the Pd lattice parameter is adjusted to the Ag parameter. The calculated Ag–Pd (111) distance at the interface is 2.31 Å. Meanwhile, the interplanar distances after the interface layers shift to 2.46 and 2.21 Å at the Ag and Pd sides, respectively (Figure 6g). Far from the interface, toward the Ag slab, a bulk-like interplanar distance is recovered. On the Pd side, the distance is 2.21 Å, 0.06 Å lower than that of pure Pd because of the lattice modification imposed by the Ag substrate.

**Figure 7:**
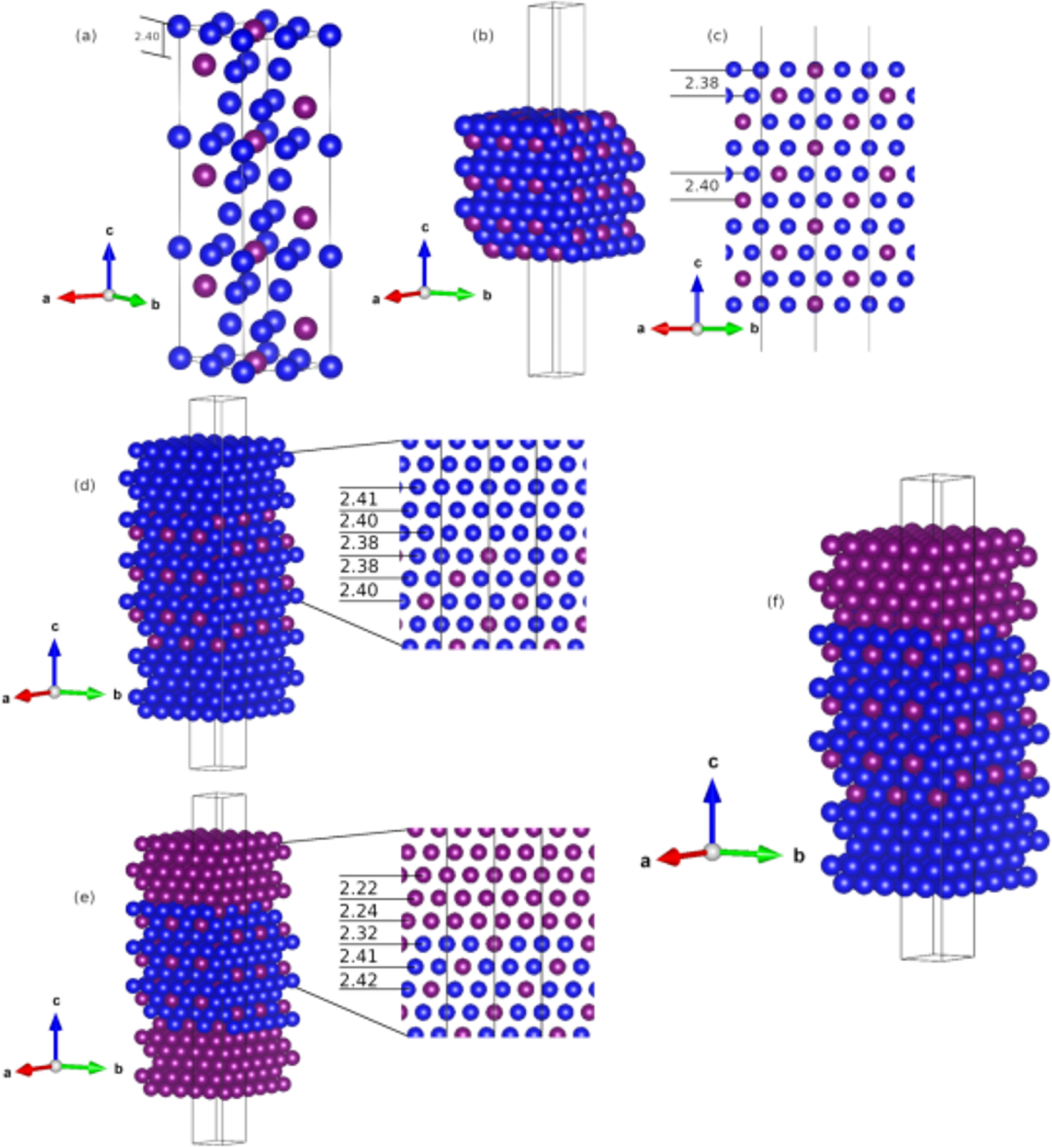
(a) Perspective view of the optimized Ag_3_Pd structure, (b) the (111) alloy surface, and (c) a transversal view with the calculated (111) interplanar distance at the surface and in the bulk. (d) Ag/AgPd model with the calculated interplanar distance near the interface, (e) Pd/AgPd model with the calculated interplanar distance near the interface, and (f) perspective view of the Ag/AgPd/Pd Janus model. Ag and Pd are represented by blue and magenta spheres, respectively.

In addition, Pd–Ag alloys form during the Pd nucleation in an environment with Ag atoms. Therefore, other Janus nanoparticles can be composed of Ag/AgPd or Ag/AgPd/Pd, where the AgPd alloy could be on one side of the particle (labeled as Ag/AgPd) or between Ag and Pd (labeled as Ag/AgPd/Pd). Considering such circumstances, to model the AgPd alloy, an Ag(111)-(2 × 2 × 3) supercell made of 36 Ag atoms was used; then, Pd atoms were systematically incorporated one at a time by atomic substitutions. Each atomic position and lattice parameter were optimized, selecting the energetically most favorable configurations in each case. Pd was included in Ag until reaching a 1:1 ratio. The stability of the AgPd alloy models was studied by the formation energy (*E*_Form_, used to obtain the relative stability of models with different atomic contents) calculated as: *E*_Form_ = (*E*_slab_ − (*n*_Ag_μ_Ag_) − (*n*_Pd_μ_Pd_))/*n*_total_, where *E*_slab_ is the total energy of each AgPd model, *n*_Ag_ and *n*_Pd_ are the numbers of Ag and Pd atoms of the AgPd alloy, μ corresponds to the chemical potential, and *n*_total_ = 36, the total number of atoms. The *E*_Form_ analysis shows that Ag_3_Pd is highly stable; at each (111) atomic layer, the Ag_3_Pd stoichiometry is retained (see Figure 7a). Therefore, we used Ag_3_Pd as the AgPd alloy to model the Ag/AgPd and Ag/AgPd/Pd Janus systems and to analyze the (111) interplanar distances.

The calculated Ag_3_Pd lattice parameter is 5.74 Å, 0.08 Å shorter than the Ag(111) lattice parameter because of the smaller atomic radius of the Pd atoms. The bimetallic alloy surface was optimized (Figure 7b), and the calculated (111) interplanar distances are 2.40 Å in the bulk phase and 2.38 Å at the surface (see Figure 7c). To explore the Janus particles that involve AgPd, the Ag/AgPd and Pd/AgPd models were first optimized by incorporating Ag or Pd slabs on the AgPd surface. Inversion symmetry was retained, as shown in Figure 7d,e. The final Ag/AgPd/Pd model is shown in Figure 7f.

## Conclusion

In this work we successfully synthesized silver–palladium Janus nanoparticles from independent sources by inert gas condensation. Different interplanar distances were obtained (0.204 and 0.230 nm) corresponding to the (111) plane of cubic Ag and the (111) plane of a cubic AgPd compound. Obtaining BNPs from metallic targets in independent chambers opens the possibility to synthesize bimetallic nanoparticles that are difficult to obtain by chemical methods. MD and electron microscopy simulations show that the features observed in the electron micrographs of real Janus AgPd BNPs can be fairly reproduced if the Ag lattice is slightly rotated with respect to the Pd lattice. Using DFT approximations, we have proposed a set of structural models that could represent the synthesized Janus particles. The suggested DFT structural models aim to understand the atomic arrangement at the interface of the Janus particle. These models are Pd epitaxially growing over Ag(111) (Ag/Pd model), the growth of Ag_3_Pd over Ag(111) (Ag/AgPd model), and both Ag and Pd connected by a Ag_3_Pd interface (Ag/AgPd/Pd model).

## Data Availability

All data that supports the findings of this study is available in the published article and/or the supporting information to this article.
